# Measles Virus Glycoprotein-Based Lentiviral Targeting Vectors That Avoid Neutralizing Antibodies

**DOI:** 10.1371/journal.pone.0046667

**Published:** 2012-10-10

**Authors:** Sabrina Kneissl, Tobias Abel, Anke Rasbach, Julia Brynza, Jürgen Schneider-Schaulies, Christian J. Buchholz

**Affiliations:** 1 Molecular Biotechnology and Gene Therapy, Paul-Ehrlich-Institut, Langen, Germany; 2 Institute of Virology and Immunobiology, University of Würzburg, Würzburg, Germany; St.Louis University, United States of America

## Abstract

Lentiviral vectors (LVs) are potent gene transfer vehicles frequently applied in research and recently also in clinical trials. Retargeting LV entry to cell types of interest is a key issue to improve gene transfer safety and efficacy. Recently, we have developed a targeting method for LVs by incorporating engineered measles virus (MV) glycoproteins, the hemagglutinin (H), responsible for receptor recognition, and the fusion protein into their envelope. The H protein displays a single-chain antibody (scFv) specific for the target receptor and is ablated for recognition of the MV receptors CD46 and SLAM by point mutations in its ectodomain. A potential hindrance to systemic administration in humans is pre-existing MV-specific immunity due to vaccination or natural infection. We compared transduction of targeting vectors and non-targeting vectors pseudotyped with MV glycoproteins unmodified in their ectodomains (MV-LV) in presence of α-MV antibody-positive human plasma. At plasma dilution 1∶160 MV-LV was almost completely neutralized, whereas targeting vectors showed relative transduction efficiencies from 60% to 90%. Furthermore, at plasma dilution 1∶80 an at least 4-times higher multiplicity of infection (MOI) of MV-LV had to be applied to obtain similar transduction efficiencies as with targeting vectors. Also when the vectors were normalized to their p24 values, targeting vectors showed partial protection against α-MV antibodies in human plasma. Furthermore, the monoclonal neutralizing antibody K71 with a putative epitope close to the receptor binding sites of H, did not neutralize the targeting vectors, but did neutralize MV-LV. The observed escape from neutralization may be due to the point mutations in the H ectodomain that might have destroyed antibody binding sites. Furthermore, scFv mediated cell entry via the target receptor may proceed in presence of α-MV antibodies interfering with entry via the natural MV receptors. These results are promising for *in vivo* applications of targeting vectors in humans.

## Introduction

For genetic modification of primary cells, lentiviral vectors (LVs) are the most suitable vectors as they stably integrate the transferred gene into the genome of dividing as well as nonproliferating cells [1]. This property has made LVs ideal gene delivery vehicles for *ex vivo* gene therapy in many ongoing clinical studies [2]. The lentiviral envelope protein can be exchanged with glycoproteins derived from other viruses (pseudotyping) and current state-of-the-art vectors are equipped with the glycoprotein of vesicular stomatitis virus (VSVG), which is very stable and allows production of vectors with high titers. Furthermore, it has a wide tropism and mediates nonselective cell entry into basically all types of mouse, rat and human cells. However, restricting cell entry to the cell population of interest is expected to improve safety and efficiency of LV mediated gene transfer and to expand its therapeutic applicability to *in vivo* gene therapy [3]. We have recently developed a flexible and highly specific targeting method for LVs, which is based on the incorporation of engineered measles virus (MV) glycoproteins into the lentiviral envelope [4]–[8]. For pseudotyping of LVs, the MV glycoproteins, namely, hemagglutinin (H) protein, responsible for receptor recognition, and fusion (F) protein, mediating membrane fusion between virus and host cell, have to be truncated in their cytoplasmic tails to allow efficient incorporation into the lentiviral membrane [4]. To interfere with cell entry via the MV receptors human CD46 and signaling lymphocyte activation molecule (SLAM), we then mutated the truncated H protein, which is derived from the NSe variant of the MV vaccine strain Edmonston B, at four residues in its ectodomain, namely Y481A, R533A, S548L and F549S [9]. The desired receptor specificity is provided by displaying a single-chain antibody (scFv) specific for the target receptor on the mutated H protein (H_mut_-scFv). This way, very different cell surface molecules including type1-membrane glycoproteins (CD105), pentaspan membrane glycoproteins (CD133), membrane tetraspan calcium channels (CD20) as well as multi-subunit ion-channels (glutamate receptors, GluR) can be used for entry by these vectors. The respective targeting vectors were not only able to selectively transduce receptor-positive cell lines, but also the corresponding target receptor-positive primary cells [4], [7]. More importantly, remarkable target specificity was observed *in vivo*, upon local or systemic injection into mice [7], [8], paving the way for *in vivo* applications in humans. For example, modification of CD105-positive endothelial cells to express blood clotting factors in hemophilia patients can only be accomplished *in vivo*. Also direct *in vivo* transduction of hematopoietic stem cells for corrections of inherited diseases would avoid costly cell isolation and *ex vivo* cell expansion. However, a potential hindrance to systemic administration of the targeting vectors in humans is pre-existing MV-specific immunity in almost all individuals due to vaccination or natural infection. The MV-protective humoral immune response is mainly directed against the H protein [10]. Rapid clearance of targeting vectors by neutralizing α-H antibodies in the plasma may therefore impair *in vivo* applications in humans.

Here, we investigated the sensitivity of targeting vectors to MV neutralizing antibodies in human plasma. We hypothesized that the displayed scFv and the mutations in the H ectodomain may sterically hinder antibody binding, hence, protect the targeting vectors against neutralization. We compared transduction efficiencies of targeting vectors and non-targeting vectors pseudotyped with MV glycoproteins that are unmodified in their ectodomains (MV-LV) in presence of α-MV antibody-positive human plasma as well as monoclonal neutralizing α-H antibodies. The data suggest that targeting vectors are partially protected against MV neutralizing antibodies.

## Results

### Transduction in presence of α-MV antibody-positive human plasma

**Figure 1:**
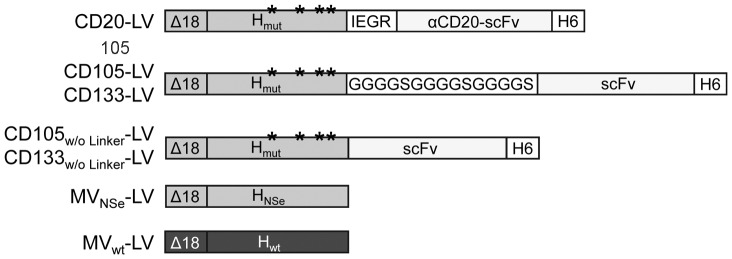
Schematic drawing of cytoplasmic tail-truncated hemagglutinin envelope proteins used for pseudotyping of lentiviral vectors. In the mutated hemagglutinin protein (H_mut_) that is derived from the NSe variant of the measles virus (MV) vaccine strain Edmonston B, mutations in the MV receptor recognition regions Y481A, R533A, S548L and F549S (ectodomain) are indicated by asterisks. Glycine-serine linker ((G_4_S)_3_) or the factor Xa cleavage site (IEGR) were used as linker region between H_mut_ and single-chain antibody (scFv). A histidine tag (H6) is present at the scFv C-terminus. The hemagglutinin protein derived from the NSe variant of the MV vaccine strain Edmonston B that is not mutated and does not display a scFv is labeled H_NSe_. The hemagglutinin protein derived from the wild-type measles virus strain IC-B is labeled H_wt_. All hemagglutinin proteins are truncated by 18 amino acids in their cytoplasmic tail (Δ18) to allow incorporation into the lentiviral envelope. The names of the respective vector particles pseudotyped with the depicted H variants are indicated on the left site. w/o: without.

The ability to transduce target cells in presence of α-MV antibody-positive human plasma was investigated for targeting vectors specific to human CD20, CD105 or CD133 (**Fig. 1**) as well as for non-targeting vectors, pseudotyped with the MV glycoproteins derived from the NSe variant of the MV vaccine strain Edmonston B (MV_NSe_-LV) or the MV wild-type strain IC-B (MV_wt_-LV, **Fig. 1**). VSVG-LV vectors, which are not affected by α-MV antibodies, were used as control.

**Figure 2:**
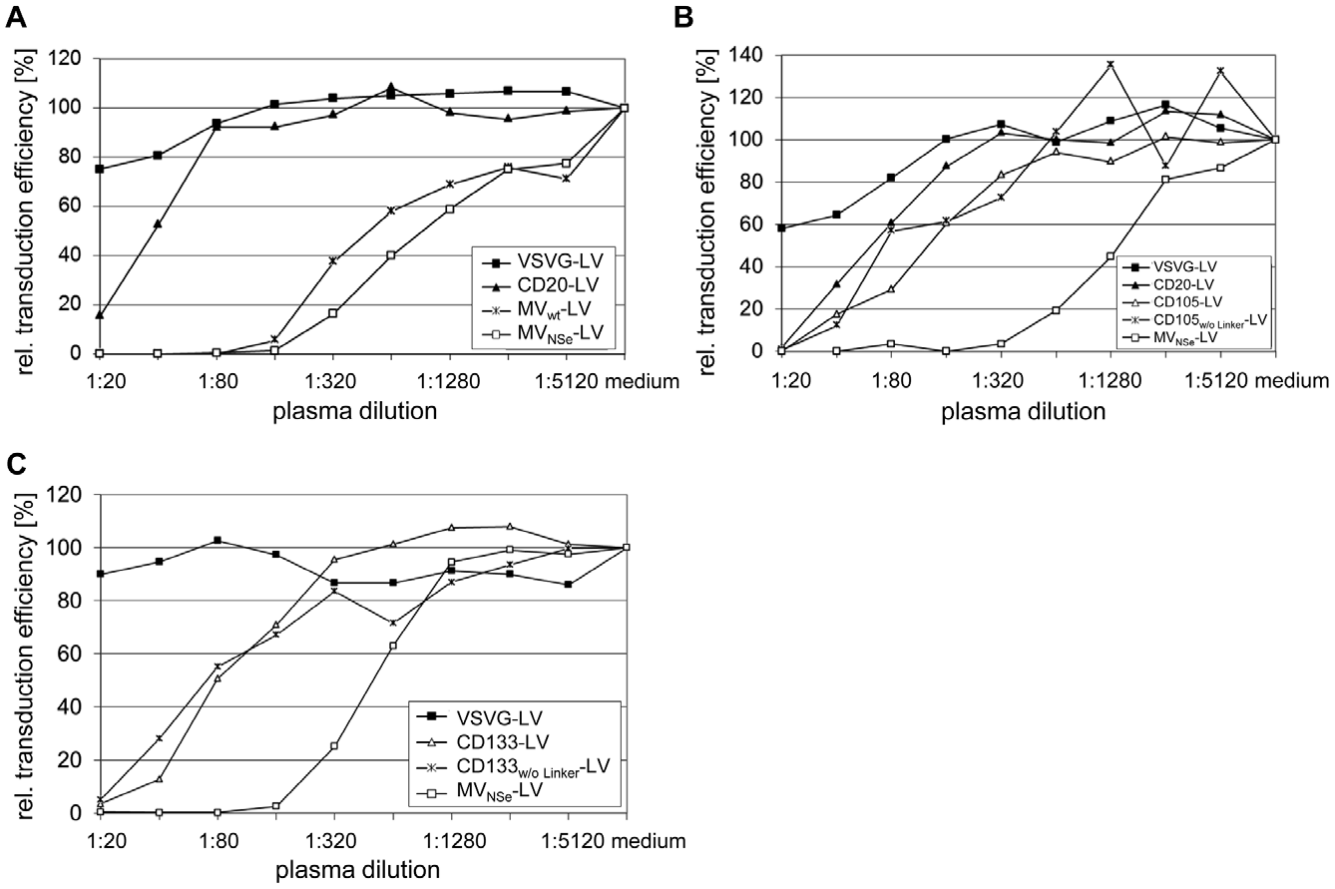
Targeting vectors are protected against MV neutralizing antibodies. The indicated vector particles were incubated in serial plasma dilutions of two different α-MV antibody-positive donors. (**a**) 3×10^4^ CD20-positive Raji cells (MOI 0.4) were added, or the dilutions were added to (**b**) CD105/CD20-positive HT1080-CD20 (MOI 0.3) or (**c**) HT1080-CD133 cells (MOI 0.3) that were seeded at a density of 1.7×10^4^ and 0.75×10^4^ cells per 96 well, respectively, 24 h before transduction. Forty-eight to 72 h later, the fraction of EGFP-positive cells was quantified by FACS analysis. The relative transduction efficiency compared to transduction in absence of plasma (medium control) of one representative donor is shown for each cell line.

Targeting vectors as well as MV_NSe_-LV, MV_wt_-LV and VSVG-LV vectors, which transfer the genetic information for the enhanced green fluorescent protein (EGFP), were incubated in serial complement inactivated α-MV antibody-positive human plasma dilutions, before the mixtures were added to the respective CD20-, CD105- or CD133-positive target cells. The relative transduction efficiencies compared to transduction in absence of plasma were determined. All the targeting vectors were substantially less sensitive to neutralization than MV_NSe_-LV and MV_wt_-LV vectors. MV_NSe_-LV was almost completely neutralized at plasma dilution 1∶160, whereas CD20-LV, CD105-LV and CD133-LV showed relative transduction efficiencies of 90%, 60% and 70%, respectively (**Fig. 2**). The CD20-LV vector showed partial protection against MV neutralizing antibodies on Raji cells (**Fig. 2a**) as well as on HT1080-CD20 cells (**Fig. 2b**), demonstrating that the observed difference between targeting vectors and MV_NSe_-LV was not cell line dependent.

Among the targeting vectors tested, CD20-LV showed the most efficient escape from neutralization that came close to that of VSVG-LV (**Figs. 2a,b**). CD20-LV differs from the other targeting vectors in the configuration of the linker connecting the αCD20-scFv with the H_mut_ protein. Whereas the αCD105- and αCD133-scFvs are fused via a long flexible (G_4_S)_3_ linker to the H_mut_ protein, a short factor Xa cleavage site (IEGR) is present in CD20-LV (**Fig. 1**). To test if the less flexible connection between H_mut_ protein and αCD20-scFv may lead to a better shielding of the H_mut_-αCD20 protein against MV neutralizing antibodies, we deleted the (G_4_S)_3_ linker in the H_mut_-αCD105 and H_mut_-αCD133 constructs. However, this did not enhance the transduction efficiency of the vectors pseudotyped with the respective H_mut_-scFv proteins (CD105_w/o Linker_-LV and CD133_w/o Linker_-LV) in presence of α-MV antibody-positive human plasma (**Figs. 2b,c**).

**Figure 3:**
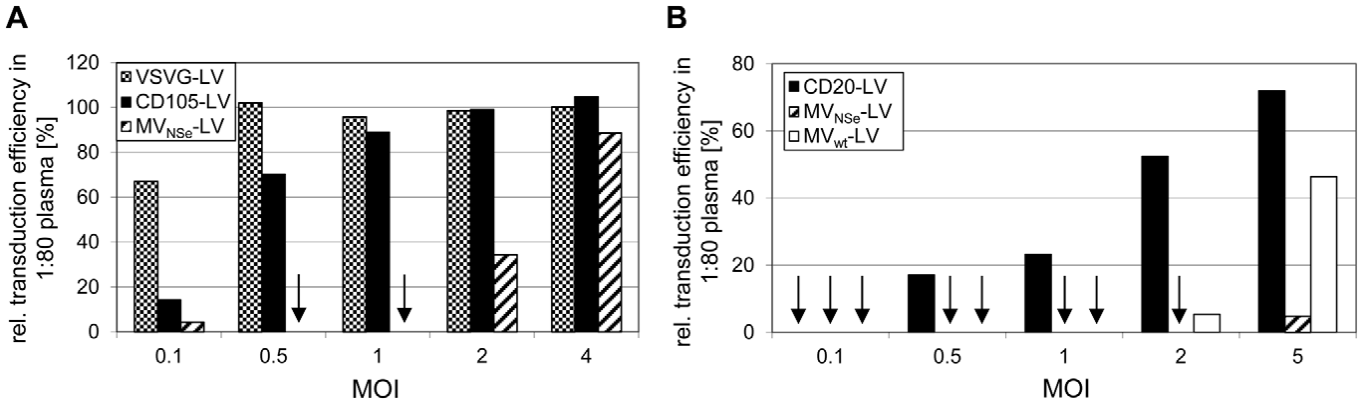
Influence of vector dose on neutralization. The indicated vector particles were incubated at varying doses in a 1∶80 plasma dilution containing (**a**) 1200 mU/ml or (**b**) 4900 mU/ml α-MV antibodies. Then, the dilutions were added to (**a**) CD105-positive HT1080-CD20 cells that were seeded at a density of 1.7×10^4^ cells per 96 well 24 h before transduction or (**b**) 3×10^4^ CD20-positive Raji cells were added to the dilutions. Forty-eight to 72 h later, the fraction of EGFP-positive cells was determined by FACS analysis. The relative transduction efficiency is shown (transduction efficiency in presence of a 1∶80 FCS dilution was set to 100%). Arrows indicate relative transduction efficiencies of <1%.

Next, we incubated varying doses of CD20-LV, CD105-LV, MV-LV and VSVG-LV, respectively, in a 1∶80 plasma dilution. We found that a 4-times higher MOI of MV_NSe_-LV had to be applied to obtain similar transduction efficiencies on target cells as with CD105-LV (**Fig. 3a**). As expected, VSVG-LV vectors were not significantly affected by the plasma. Compared to CD20-LV, even a 5-times higher MOI of MV_NSe_-LV was not sufficient to obtain similar transduction efficiencies in the presence of 1∶80 plasma (**Fig. 3b**). Hence, more infectious particles are needed for MV_NSe_-LV and MV_wt_-LV (**Fig. 3b**) than for targeting vectors to allow transduction in the presence of MV neutralizing antibodies. We observed in several experiments that plasma from donors with a high α-MV antibody titer led to stronger neutralization compared to plasma with a lower titer (data not shown), which explains why all vectors in the experiment depicted in **Fig. 3b** were generally stronger neutralized than the ones shown in **Fig. 3a**.

### Neutralization escape of targeting vectors using equal physical particle numbers

**Figure 4:**
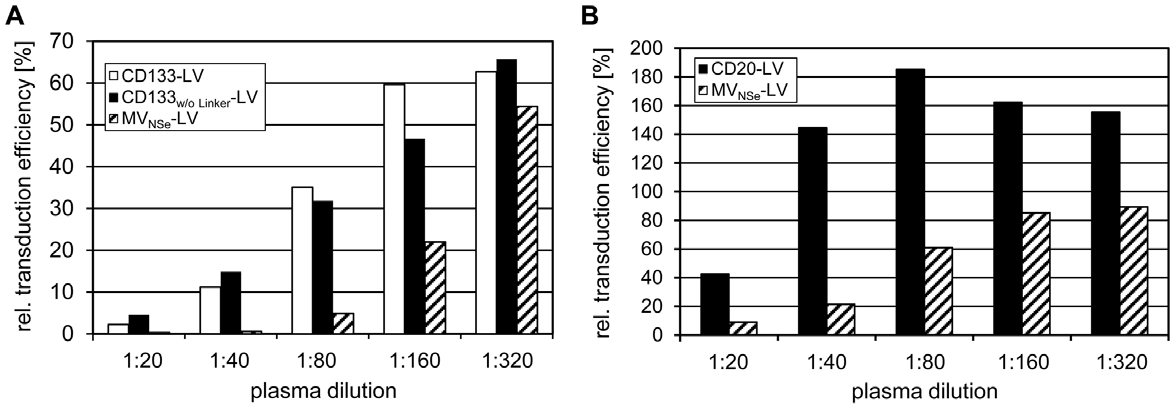
Influence of particle amount on neutralization. Equal amounts of vector particles as determined by p24 ELISA were incubated in serial plasma dilutions of two different α-MV antibody-positive donors. The dilutions were added to (**a**) CD133-positive HuH7 cells that were seeded 24 h before transduction at a density of 1.0×10^4^ (CD133-specific vectors) and 5.0×10^4^ (MV_NSe_-LV) cells per 96 well, respectively, to apply similar MOIs. Alternatively, the dilutions were added to (**b**) 1.0×10^4^ (CD20-LV) and 5.0×10^4^ (MV_NSe_-LV) CD20-positive Raji cells, respectively. Seventy-two h later, the percentage of EGFP-positive cells was determined by FACS analysis. The relative transduction efficiency compared to transduction in absence of plasma of one representative donor is shown.

In comparison to MV_NSe_-LV vectors, the ratio of infectious to non-infectious particles is similar for CD105-LV, but on average 2–10-fold lower for CD133- and CD20-targeting vectors [7]. Hence, by applying identical MOIs, more physical particles of the two targeting vectors have been used. We therefore incubated equal particle numbers of MV_NSe_-LV, CD20-LV and CD133-targeting vectors, as determined by p24 assay, in serial dilutions of α-MV antibody-positive human plasma. Transduction efficiencies were then determined on CD133-positive HuH7 cells or CD20-positive Raji cells. For a reliable read-out, transduction rates should be between 10% and 50%. Above 50%, neutralization might be underestimated due to multiple vector integrations, below 10% small background variations might be overestimated when calculating the relative transduction efficiency. Therefore, cells were seeded at an optimal density for MV_NSe_-LV and the targeting vectors, respectively. Using this experimental set up, CD133-specific targeting vectors were still less sensitive to α-MV antibody-positive human plasma than MV_NSe_-LV (**Fig. 4a**), although escape was not as efficient as in the experiment depicted in **Fig. 2c**. Hence, maybe a higher number of physical particles was in part responsible for the neutralization escape of targeting vectors, but other factors than antibody saturation must also play a role. Also CD20-LV showed considerable escape, after normalizing its p24 level to that of MV_NSe_-LV (**Fig. 4b**).

### Transduction in presence of α-MV antibody-negative human serum

**Figure 5:**
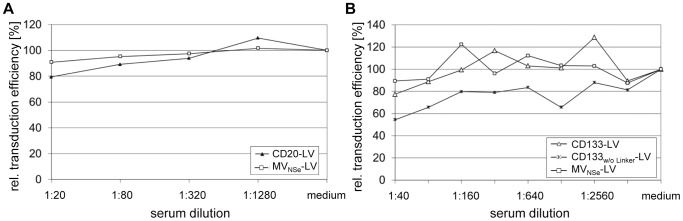
α-MV antibody-negative serum does not neutralize MV_NSe_-LV or targeting vectors. Equal amounts of physical particles of the indicated vector types were incubated in serial serum dilutions of an α-MV antibody-negative donor. Then, (**a**) 1×10^4^ (CD20-LV) and 2×10^5^ (MV_NSe_-LV) CD20-positive Raji cells were added, respectively, or the dilutions were added to (**b**) CD133-positive HuH7 cells. These were seeded at a density of 1.0×10^4^ (targeting vectors) and 5.0×10^4^ (MV_NSe_-LV) cells per 96 or 48 well, respectively, 24 h before transduction, to apply similar MOIs of vector particles. Forty-eight to 72 h later, the percentage of EGFP-positive cells was determined by FACS analysis. As control, medium without serum was used.

To verify that the observed differences between targeting vectors and MV_NSe_-LV were due to MV neutralizing antibodies in human plasma, we incubated CD20-LV, CD133-LV, CD133_w/o__Linker_-LV and MV_NSe_-LV vectors in serial dilutions of complement inactivated α-MV antibody-negative human serum from non-vaccinated donors without previous MV infection. Also in this experiment same p24 levels and similar MOIs were applied. As expected, the α-MV antibody-free serum did not neutralize the targeting vectors or MV_NSe_-LV (**Fig. 5**). It had no significant influence on transduction efficiency. This verifies that the observed differences between targeting vectors and MV_NSe_-LV were due to α-MV antibodies in human plasma and an at least partial resistance of targeting vectors to these antibodies.

### Transduction in presence of MV neutralizing monoclonal antibodies

**Figure 6:**
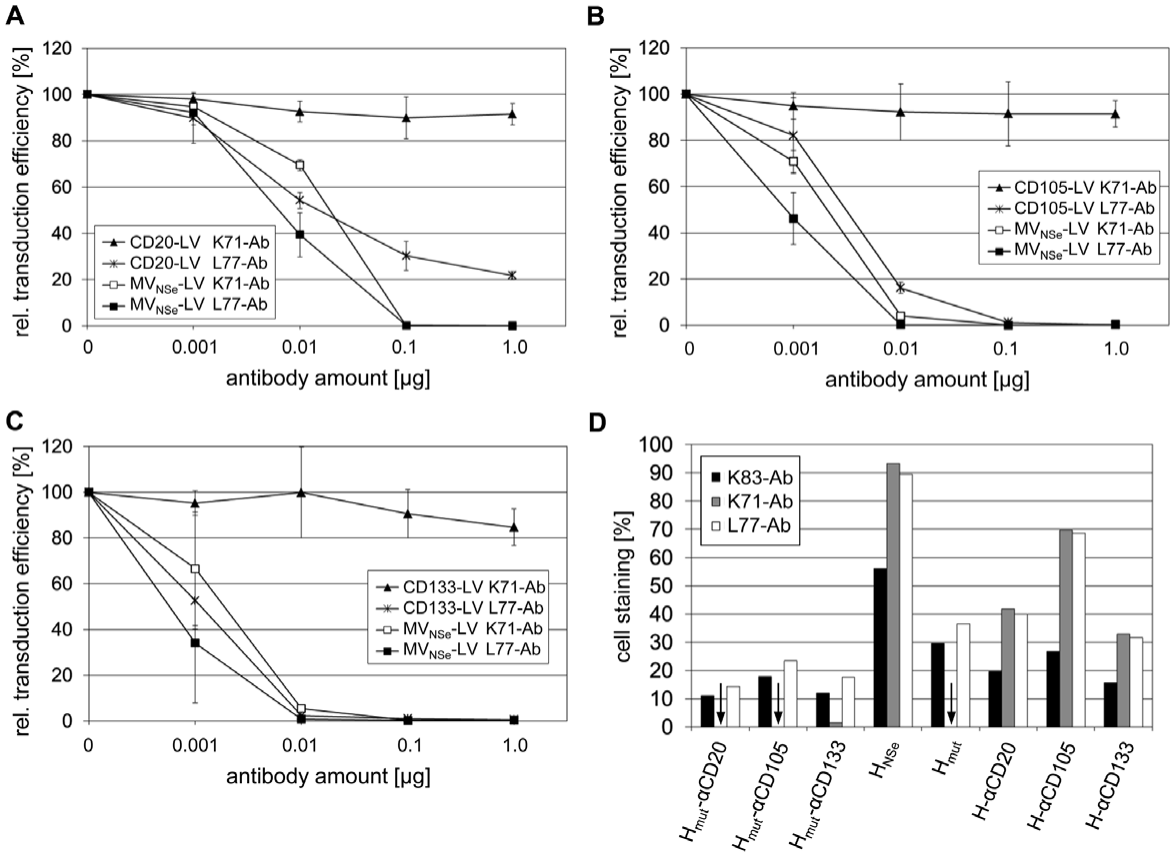
Targeting vectors are protected against the neutralizing antibody K71. Equal amounts of physical particles of the indicated vector types were incubated in presence of increasing amounts of antibody K71 (K71-Ab; putative epitope near the mutation sites in H_mut_-scFv constructs) and L77 (L77-Ab; putative epitope distant to the mutation sites in H_mut_-scFv constructs), respectively, in a final volume of 100 µl. After incubation at room temperature for 1 h, (**a**) 3×10^4^ (CD20-LV) or 4.5×10^5^ (MV_NSe_-LV) CD20-positive Raji cells were added, or the vectors were added to (**b**) 9.3×10^4^ (CD105-LV) or 3.4×10^4^ (MV_NSe_-LV) CD105-positive HT1080 cells or (**c**) 3.4×10^4^ (CD133-LV) or 2.7×10^4^ (MV_NSe_-LV) CD133-positive HuH7 cells, to apply an MOI of 0.25 for each vector type. Forty-eight hours later, the fraction of EGFP-positive cells was quantified by FACS analysis. Mean values (n = 3) and s.d. of the relative transduction efficiency compared to transduction in absence of antibody is shown for each cell line. (**d**) The indicated H proteins were expressed on the surface of HEK-293T cells. The control antibody K83 and the antibodies K71 and L77 were incubated with the cells, respectively, and a FITC-labeled secondary antibody was used to detect antibody binding to the different H proteins by FACS analysis. The percentage of FITC-positive cells subtracted by the staining of secondary antibody alone is shown. Arrows indicate 0% cell staining.

To evaluate if the mutations present in the H_mut_-scFv constructs to ablate recognition of the natural MV receptors are responsible for the neutralization escape of the targeting vectors, we incubated CD20-LV, CD105-LV, CD133-LV and MV_NSe_-LV vectors with increasing amounts of the monoclonal neutralizing α-H antibodies K71 and L77, respectively, and added the mixtures to the respective target cells. For K71 escape mutations close to the CD46 and SLAM binding sites in H have been identified, whereas the escape mutations of L77 are more distant to the receptor binding sites [11], [12]. MV_NSe_-LV was entirely neutralized when 0.1 µg of K71 was applied. In contrast, the three targeting vectors escaped neutralization by K71 almost completely (**Figs. 6a–c**). Using 0.1 µg L77 antibody, MV_NSe_-LV as well as CD105-LV and CD133-LV vectors were fully neutralized and CD20-LV showed a reduced relative transduction efficiency of 30% (**Figs. 6a–c**). Next, we expressed the H proteins used for pseudotyping on the surface of HEK-293T cells. In parallel, we expressed the mutated H protein not displaying a scFv (H_mut_) and H proteins that were not blinded for the MV receptors, but displayed the αCD20-, αCD105- or αCD133-scFv (H-scFv) in HEK-293T cells. The cells were then incubated with the antibodies K71 or L77 and a FITC-conjugated secondary antibody and were analyzed by FACS. As positive control, the primary antibody K83 [11] was used, which was previously shown to bind the H protein [6]. K83 and L77 bound H_mut_-αCD20, H_mut_-αCD105, H_mut_-αCD133 and H_mut_ with similar efficiency, whereas K71 binding was greatly reduced. In contrast, both K71 and L77 efficiently bound H_NSe_ and the different H-scFv proteins, lacking the mutation sites (**Fig. 6d** **, Fig. S1**). Hence, the neutralization escape of the targeting vectors from the K71 antibody is likely due to the inability of the antibody to efficiently bind to H_mut_-scFv constructs.

## Discussion

Targeting vectors are especially suited for *in vivo* administrations as they are specific for their target cell populations, leaving other cells unmodified. The targeting vectors investigated here are pseudotyped with engineered MV glycoproteins displaying scFvs specific for cell surface receptors of interest. Point mutations in the H protein ectodomain ablate recognition of the native MV receptors CD46 and SLAM. The recently identified third MV receptor, which was found to be nectin-4 [13], [14], and to mediate MV infection of lung epithelial cells, played no role in this study as all cells used were nectin-4 negative (data not shown).

We demonstrate here that lentiviral targeting vectors pseudotyped with engineered MV glycoproteins are partially protected against α-MV antibodies in human plasma.

As most MV neutralizing antibodies are directed against H and not F protein [10], it is most likely that the modifications introduced in H for receptor targeting reduced antibody binding. Due to the extensive glycosylation of the H protein, the antibody response is mostly directed towards a limited number of immunodominant domains including the receptor binding sites, with a strong antibody response to the SLAM and a weaker response to the CD46 recognition site [15], [16]. Both regions are mutated in the H protein used for lentiviral retargeting. These mutations might have destroyed the epitopes of neutralizing antibodies interfering with receptor recognition. This hypothesis is supported by the results depicted in Figure 6. Whereas the K71 antibody was not able to neutralize the targeting vectors, the L77 antibody neutralized all vector types. Based on escape mutants of the MV strain CAM/RB, amino acid residues conferring escape from the antibodies K71 (E492K, S550P) and L77 (P377Q, M378K) had been identified [11], [12]. Amino acid residues S550 and M378 are also present in the H protein derived from the Edmonston B strain used in this study. In the H protein crystal structure [16], amino acid residue S550 is located close to the mutations R533A, S548L and F549S present in H_mut_ and H_mut_-scFv to render them deficient for use of the natural MV receptors. In contrast, residue M378, which is important for L77 binding, is distant to all four mutations. Hence, the epitope of K71 is likely destroyed in H_mut_-scFv constructs, preventing binding of the antibody. The observation that K71 can bind the H-scFv constructs, but not the H_mut_ protein further supports this hypothesis (**Fig. 6d** **, Fig. S1**). Recently, Lévy et al. [17] demonstrated that MV_NSe_-LV vectors that efficiently transduce resting lymphocytes [18], [19], representing promising candidates for *in vivo* applications, could be protected *in vitro* against monoclonal neutralizing antibodies by introducing mutations into the Noose and NE epitopes in H. These epitopes form beside the receptor recognition regions further binding sites for neutralizing antibodies [20], [21]. Interestingly, an additional glycosylation site at position N416, which is present in newly emerged MV-D genotypes, was required to gain also protection against α-MV antibody-positive human serum [17]. Probably the introduced glycosylation sterically hinders binding of neutralizing antibodies. Likewise, besides the introduced mutations, the displayed scFv on H_mut_-scFv protein in targeting vectors may contribute to protection against neutralizing antibodies in human plasma. In the absence of structural information about the H protein C-terminus [22], its exact position relative to H protein cannot be predicted. It is however likely that the flexible H protein C-terminus allows the scFv to sterically hinder neutralizing antibody binding not only in the receptor recognition regions but also in other sites. Finally, it is conceivable that effective neutralization of the vectors is difficult in the absence of antibodies directed against the scFv itself. Such antibodies are expected to efficiently prevent target receptor binding and scFv mediated cell entry.

Interestingly, CD20-LV escaped neutralization more efficiently than CD105-LV and CD133-LV. We excluded that the higher flexibility of the linker peptide between H_mut_ protein and αCD105- or αCD133-scFv influenced neutralization escape (**Figs. 2b,c**). Differences in the scFv framework and thus the scFv 3D structure could in principle explain subtle differences in neutralization escape. However, the framework regions between the three scFvs, especially between αCD133- and αCD20-scFvs, are very similar. The CD133- and CD20-specific scFvs are both of murine origin, the heavy chains are even derived from the same germ line. Alternatively, receptor topology may have an influence on neutralization as all three target receptors belong to different protein classes: CD20 is a membrane tetraspan calcium channel, whereas CD105 is a type1-membrane glycoprotein and CD133 a pentaspan membrane glycoprotein. Possibly, the epitope on CD20 is better accessible for the αCD20-scFv even when antibodies are bound to H protein than the epitopes for the CD133- and CD105-specific scFv. This hypothesis is in line with our observation that only CD20-LV escaped neutralization by L77 to a certain extent, although L77 bound H_mut_-αCD20 as efficient as H_mut_-αCD105 and H_mut_-αCD133 (**Figure 6**).

We demonstrate here for three different targeting vectors that they are all partially protected against MV neutralizing antibodies in human plasma. Thus, our data indicate that targeting vectors in general can escape neutralization by α-MV antibodies at least to a certain extent, which suggests that single systemic applications may be possible despite pre-existing α-MV immunity. For repeated cycles of *in vivo* vector administration, pseudotyping of LVs with engineered H and F glycoproteins of other paramyxoviruses, such as the Tupaia paramyxovirus (TPMV) is an option [23].

## Materials and Methods

### Ethics Statement

The research has been approved by the Ethics Committee of the University Hospital Frankfurt, Germany. Written informed consent was obtained from each donor.

### Plasmid construction

The plasmids encoding the truncated H_NSe_, H_wt_ or H_mut_ protein as well as the plasmids encoding the truncated H_mut_ protein displaying the αCD20-scFv via the factors Xa site (pCG-H_mut_-αCD20) or the αCD105-scFv and αCD133-scFv via a (G_4_S)_3_ linker (**Fig. 1**), respectively, were described previously [4], [6], [7]. For generation of constructs pCG-H_mut_-αCD105 and pCG-H_mut_-αCD133 without (G_4_S)_3_ linker, the respective scFv coding regions were cloned into the SfiI and NotI digested pCG-H_mut_-αCD20 backbone thereby replacing the factor Xa-αCD20-scFv coding region in pCG-H_mut_-αCD20. For generation of the plasmids pCG-H-αCD20, pCG-H-αCD105 and pCG-H-αCD133, the scFv coding regions were cloned into the SfiI and NotI digested pCG-H_NSe_Δ18His backbone.

The H protein plasmids were purified with the EndoFree® Plasmid Maxi Kit (Qiagen, Hilden, Germany), whereas all other plasmids used for vector particle production were purified by the PlasmidFactory GmbH & Co. KG, Bielefeld, Germany.

### Cultivation of cell lines

The culture of HEK-293T, Raji, HT1080 and HT1080-CD20 cells has been described previously [4]. HT1080 cells stably transfected with a CD133 expression plasmid (HT1080-CD133) [7] were cultured in Dulbecco's modified Eagle medium (DMEM) supplemented with 10% FCS, 2 mM L-glutamine and 0.5 mg/ml G418. HuH7 cells (JCRB cell bank) were cultured in DMEM supplemented with 10% FCS and 2 mM L-glutamine.

### Vector particle production and titration

Lentiviral vector particles were produced by polyethyleneimine (PEI) based transfection of HEK-293T cells. For this purpose, 2×10^7^ cells were seeded 24 h before transfection into a T175 flask. On the day of transfection, medium was replaced with 10 ml of DMEM supplemented with 15% FCS and 2 mM L-glutamine. For targeting vectors, 1.35 µg of MV H_mut_-scFv protein expression plasmid, 4 µg of pCG-FcΔ30 [4], 14.4 µg of packaging plasmid pCMVΔR8.9 [24] and 15.2 µg of transfer vector plasmid pSEW [25], encoding the enhanced green fluorescent protein (EGFP), were mixed with 2.3 ml DMEM without supplements. For MV-LV vectors, 1.35 µg of MV H_NSe_ protein or 4 µg MV H_wt_ protein expression plasmid and 9.4 µg or 6.7 µg of pCG-FcΔ30, respectively, together with 9.1 µg pCMVΔR8.9 and 15.2 µg pSEW were mixed with 2.3 ml DMEM without supplements. In parallel, 140 µl of 18 mM PEI and 2.2 ml DMEM without supplements were mixed. The PEI mixture and the DNA mixture were combined, vortexed and incubated for 20 min at room temperature (25°C), before the transfection mixture was added to the cells. Twenty-four h later, medium was exchanged against 16 ml fresh DMEM supplemented with 10% FCS and 2 mM L-glutamine. The cell supernatant containing the pseudotyped vector particles was filtered 24 h later (0.45-µm filter) and concentrated by ultracentrifugation over a 20% (wt/vol) sucrose cushion (100 000 g for 3 h at 4°C). The supernatant was discarded and the pellet was resuspended in 60 µl PBS. Vector particles pseudotyped with VSVG were produced by co-transfection of 6.13 µg pMD.G2 (kindly provided by Didier Trono, Tronolab, Lausanne, Switzerland), 11.4 µg pCMVΔR8.9 and 17.5 µg pSEW. Targeting vectors and control vectors were titrated together on the same cell line used for the neutralization assays. EGFP-positive cells were determined by FACS analysis and vector stock titers were calculated as described previously [4], [7]. The p24 values of the vector preparations were determined using the HIV-1 p24 antigen ELISA RETROtek kit (ZeptoMetrix Corporation, Buffalo, NY, USA).

### Expression of H protein variants in HEK-293T cells

1×10^6^ HEK-293T cells were seeded in each well of a 6 well plate. Twenty-four hours later, the cells were transfected by Lipofectamine and Plus Reagent (Invitrogen, Darmstadt, Germany) with 3 µg per well of the plasmids encoding the different H protein variants or the empty control plasmid pCG-1 according to the manufacturer's instructions. After 24 h, the cells were used for antibody staining and FACS analysis.

### Neutralization assays

Human plasma was collected from MV vaccinated healthy donors using the BD Vacutainer™ CPT™ system with sodium citrate (BD, Heidelberg, Germany). The MV antibody titers of all donors were in the range of 1200–4900 mU/ml. α-MV antibody-negative serum was collected from healthy non-MV vaccinated donors without previous MV infection. All samples were complement inactivated by incubation at 56°C for 30 min. For antibody binding, the indicated vector particles were incubated in a 96 well plate for 20 min at 4°C in serial dilutions of plasma or serum. Before adding the dilutions to the respective cell lines, they were pre-warmed at 37°C for 5–10 min. Alternatively, the indicated vector particles were incubated for 1 h at RT in presence of different concentrations of the neutralizing α-H antibody K71 or L77 [11], [12], before they were added to the cells. Plates were incubated for 3 h at 37°C before an exchange with fresh medium was performed. Forty-eight to 72 h later, the percentage of EGFP-positive cells was determined by FACS analysis. The relative transduction efficiency compared to transduction in absence of plasma/serum/antibody was determined. In another experiment, a constant plasma dilution was used (1∶80) and the amount of incubated vector particles was varied. As control, vector particles were incubated in parallel in a 1∶80 dilution of complement inactivated FCS. The relative transduction efficiency compared to transduction in presence of FCS was determined.

### Flow cytometry

Flow cytometry was performed as described previously [4]. Briefly, adherent cells were detached by incubation with 50–500 µl PBS-trypsin solution. Trypsin digestion was stopped by adding 200–1000 µl medium containing 10% FCS. Cells in suspension were then transferred into Micronic tubes (Micronic Europe B.V., Lelystad, The Netherlands) and after the addition of 600 µl FACS washing buffer (PBS, 1% FCS, 0.1% NaN_3_), they were pelleted at 310 g for 3 min and 4°C. The cells were washed once more in 900 µl FACS washing buffer and finally resuspended in 100 µl PBS/1% paraformaldehyde. Alternatively, the cells were incubated after the second washing step for 1 h with the antibody K83 (1∶10), K71 (1 µg) or L77 (1 µg) [11], [12], respectively. After two additional washing steps, they were incubated for 20 min with the FITC-labeled goat polyclonal secondary antibody to mouse IgG-Fc (1∶50, Abcam plc, Cambridge, UK), before they were washed twice and finally fixed in 100 µl PBS/1% paraformaldehyde. Data were obtained with the LSR II FACS machine (BD, Heidelberg, Germany) and analysed with the FACSDiva software (BD, Heidelberg, Germany). Gates were set such that they contained less than 1% of the cells from the negative control cell population, which were untransduced cells.

## Supporting Information

Figure S1 **K71 antibody does not bind H_mut_ and H_mut_-scFv proteins.** The indicated H proteins were expressed on the surface of HEK-293T cells. As control, HEK-293T cells transfected with the empty expression plasmid pCG-1 were used. The control antibody K83 and the antibodies K71 and L77 were incubated with the cells, respectively, and a FITC-labeled secondary antibody was used to detect antibody binding to the different H proteins. The percentage of FITC-positive cells was determined by FACS analysis. When cells were incubated with the secondary mouse IgG-Fc specific antibody alone, it bound unspecifically to the displayed scFv on the H proteins, which was defined as background binding.
(TIF)
