# Supplementary figures and images for: Measles Virus Glycoprotein-Based Lentiviral Targeting Vectors That Avoid Neutralizing Antibodies
Source: PLoS One. 2012 Oct 10;7(10):e46667. doi: 10.1371/journal.pone.0046667 (PMC3468630; doi:10.1371/journal.pone.0046667)

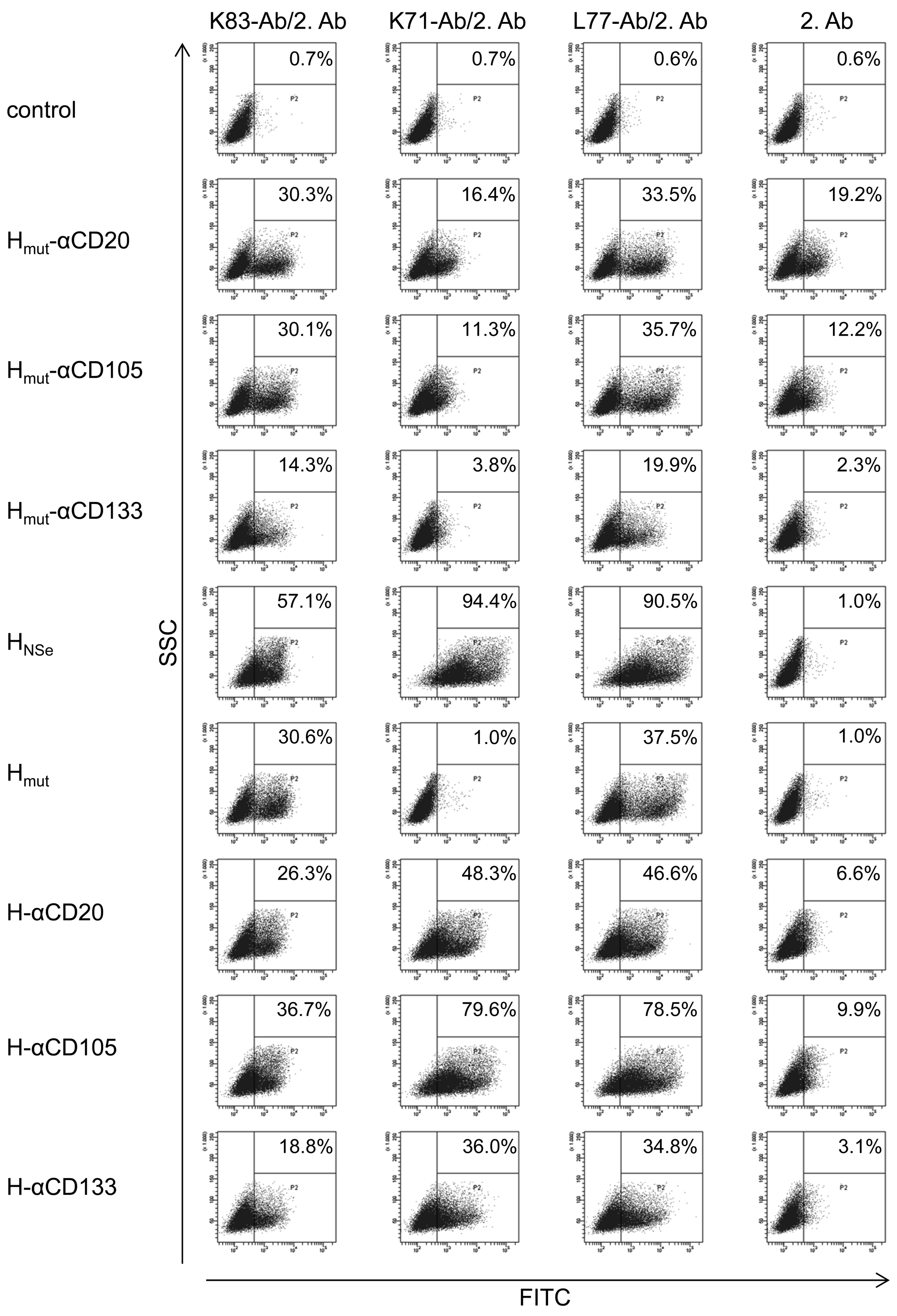

Supplement: Figure S1 — K71 antibody does not bind Hmut and Hmut-scFv proteins. The indicated H proteins were expressed on the surface of HEK-293T cells. As control, HEK-293T cells transfected with the empty expression plasmid pCG-1 were used. The control antibody K83 and the antibodies K71 and L77 were incubated with the cells, respectively, and a FITC-labeled secondary antibody was used to detect antibody binding to the different H proteins. The percentage of FITC-positive cells was determined by FACS analysis. When cells were incubated with the secondary mouse IgG-Fc specific antibody alone, it bound unspecifically to the displayed scFv on the H proteins, which was defined as background binding. (TIF) [file pone.0046667.s001.tif]
